# Supplementary material for: Yeast Eps15-like endocytic protein Pan1p regulates the interaction between endocytic vesicles, endosomes and the actin cytoskeleton
Source: eLife. 2016 Feb 25;5:e10276. doi: 10.7554/eLife.10276 (PMC4775215; doi:10.7554/eLife.10276)
Supplement: Supplementary file 1. — DOI: http://dx.doi.org/10.7554/eLife.10276.023 [file elife-10276-supp1.docx]

**Supplementary file 1**. Yeast Strains used in this study

Strain Genotype Source

DDY2544 *Mat***a** *his3*-Δ*200* *leu2-3*, *112* *ura3-52* *ark1*Δ::*HIS3 prk1*Δ::*URA3* Drubin Lab

DDY2859 *Mat***a** *his3*-Δ*200* *leu2-3*, *112* *ura3-52* *pan1*Δ::*PAN1*::*LEU2*  Drubin Lab

DDY2862 *Mat***a** *his3*-Δ*200* *leu2-3*, *112* *ura3-52 lys2-801* *pan1*Δ::*pan1-15TA*::*LEU2* Drubin Lab

DDY2873 *Mat***a** *his3*-Δ*200* *leu2-3*, *112* *ura3-52* *bar1*Δ::*LEU2* Drubin Lab

DDY2875 *Mat***a** *his3*-Δ*200* *leu2-3*, *112* *ura3-52* *pan1*Δ::*pan1-15TA*::*LEU2 bar1*Δ::*URA3* Drubin Lab

JJTY0092 *Mat***a** *his3*-Δ*200* *leu2-3*, *112* *ura3-52* *bar1*Δ::*LEU2 ark1*Δ::*HIS3 prk1*Δ::*URA3* Toshima lab

JJTY0369 *Mat***a** *his3*-Δ*200* *leu2-3*, *112* *ura3-52 lys2-801* Toshima lab

JJTY0509 *Mat***a** *his3*-Δ*200* *leu2-3*, *112* *ura3-52* *Pan1-GFP*::*HIS3* Toshima lab

JJTY0463 *Mat***a** *his3*-Δ*200* *leu2-3*, *112* *ura3-52* *Pan1-GFP*::*HIS3 ABP1-mCherry::URA3* This study

JJTY0888 *Mat***α** *his3*-Δ*200* *leu2-3*, *112* *ura3-52* *lys2-801* *pan1*Δ::*pan1-18TA*::*LEU2* This study

JJTY0889 *Mat***a** *his3*-Δ*200* *leu2-3*, *112* *ura3-52* *lys2-801* *pan1*Δ::*pan1-18TA*::*LEU2 bar1*Δ::*URA3*  This study

JJTY1162 *Mat***a** *his3*Δ*1* *leu2*Δ*0* *ura3*Δ*0* *PAN1-mCherry*::*URA3 ABP140-3GFP:: HIS3*  This study

JJTY1326 *Mat***a** *his3*-Δ*200* *leu2-3*, *112* *ura3-52* *lys2-801 HSE1-3GFP::HIS3 VPS26-3mCherry::URA3* This study

JJTY1350 *Mat***a** *his3*-Δ*200* *leu2-3*, *112* *ura3-52* *lys2-801* *pan1*Δ::*pan1-18TA*::*LEU2 ABP1-mCherry::URA3*

*VPS15-GFP::HIS3* This study

JJTY1353 *Mat***a** *his3*-Δ*200* *leu2-3*, *112* *ura3-52* *lys2-801* *pan1*Δ::*pan1-18TA*::*LEU2 ABP1-mCherry::URA3*

*VPS4-GFP::HIS3* This study

JJTY1354 *Mat***a** *his3*-Δ*200* *leu2-3*, *112* *ura3-52* *lys2-801* *pan1*Δ::*pan1-18TA*::*LEU2 ABP1-mCherry::URA3*

*EAR1-GFP::HIS3* This study

JJTY1639 *Mat***a** *his3*-Δ*200* *leu2-3*, *112* *ura3-52* *lys2-801* *pan1*Δ::*pan1-18TA*::*LEU2 bar1Δ::URA3::URA3*

*ABP1-GFP*::*HIS3* This study

JJTY1647 *Mat***a** *his3*-Δ*200* *leu2-3*, *112* *ura3-52* *lys2-801* *pan1*Δ::*pan1-18TA*::*LEU2 ABP1-mCherry::URA3*

*GPF-VPS21::HIS3* This study

JJTY1649 *Mat***a** *his3*-Δ*200* *leu2-3*, *112* *ura3-52* *lys2-801* *pan1*Δ::*pan1-18TA*::*LEU2 ABP1-mCherry::URA3*

*VPS26-3GPF::HIS3* This study

JJTY2637  *Mat***a** *his3*-Δ*200* *leu2-3*, *112* *ura3-52* *lys2-801* *pan1*Δ::*pan1-18TA*::*LEU2 ABP1-mCherry::URA3*

*GFP-SEC4*::*HIS* This study

JJTY2639 *Mat***a** *his3*-Δ*200* *leu2-3*, *112* *ura3-52* *lys2-801* *pan1*Δ::*pan1-18TA*::*LEU2 ABP1-mCherry::URA3*

*VPS52-GPF*::*HIS3* This study

JJTY2641 *Mat***a** *his3*-Δ*200* *leu2-3*, *112* *ura3-52* *lys2-801* *pan1*Δ::*pan1-18TA*::*LEU2 ABP1-mCherry::URA3*

*VPS8-GPF*::*HIS3* This study

JJTY2643  *Mat***a** *his3*-Δ*200* *leu2-3*, *112* *ura3-52* *lys2-801* *pan1*Δ::*pan1-18TA*::*LEU2 ABP1-mCherry::URA3*

*VPS24-GPF*::*HIS3* This study

JJTY2645 *Mat***a** *his3*-Δ*200* *leu2-3*, *112* *ura3-52* *lys2-801* *pan1*Δ::*pan1-18TA*::*LEU2 ABP1-mCherry::URA3*

*VPS11-GPF*::*HIS3* This study

JJTY3251 *Mat***a** *his3*-Δ*200* *leu2-3*, *112* *ura3-52* *lys2-801* *pan1*Δ::*pan1-18TA*::*LEU2 ABP1-mCherry::URA3*

*GFP-VPS36::HIS3* This study

JJTY3537 *Mat***a** *his3*-Δ*200* *leu2-3*, *112* *ura3-52* *lys2-801* *pan1*Δ::*pan1-18TA*::*LEU2 ABP1-mCherry::URA3*

*SEC7-GFP::HIS* This study

JJTY3538 *Mat***a** *his3*-Δ*200* *leu2-3*, *112* *ura3-52* *lys2-801* *pan1*Δ::*pan1-18TA*::*LEU2 ABP1-mCherry::URA3*

*HSE1-3GFP::HIS3*  This study

JJTY3539 *Mat***α** *his3*-Δ*200* *leu2-3*, *112* *ura3-52* *lys2-801* *pan1*Δ::*pan1-18TA*::*LEU2 ABP1-mCherry::URA3*

*MVB12-3GFP::HIS3* This study

JJTY4097 *Mat*α *his3*-Δ*200* *leu2-3*, *112* *ura3-52 lys2-801 HSE1-3GFP::HIS3 ABP140-tdTomato::URA*3 This study

JJTY4099 *Mat***a** *his3*-Δ*200* *leu2-3*, *112* *ura3-52* *lys2-801* *pan1*Δ::*pan1-18TA*::*LEU2 ABP1-mCherry::URA3*

*HSE1-3GFP::HIS3 las17*Δ*::KanMX* This study

JJTY4100 *Mat*α *his3*-Δ*200* *leu2-3*, *112* *ura3-52 lys2-801* *pan1*Δ::*pan1-18TA*::*LEU2 ABP1-mCherry::URA3*

*HSE1-3GFP::HIS3 bbc1*Δ::*KanMX* This study

JJTY4101 *Mat*α *his3*-Δ*200* *leu2-3*, *112* *ura3-52 lys2-801* *pan1*Δ::*pan1-18TA*::*LEU2 ABP1-mCherry::URA3*

*HSE1-3GFP::HIS3 cap1*Δ::*KanMX* This study

JJTY4103 *Mat***a** *his3*-Δ*200* *leu2-3*, *112* *ura3-52* *lys2-801* *pan1*Δ::*pan1-18TA*::*LEU2 ABP1-mCherry::URA3*

*HSE1-3GFP::HIS3 sla1* Δ*::KanMX* This study

JJTY4104 *Mat***a** *his3*-Δ*200* *leu2-3*, *112* *ura3-52* *lys2-801* *pan1*Δ::*pan1-18TA*::*LEU2 ABP1-mCherry::URA3*

*HSE1-3GFP::HIS3 sla2* Δ*::KanMX* This study

JJTY4105  *Mat***a** *his3*-Δ*200* *leu2-3*, *112* *ura3-52* *lys2-801* *pan1*Δ::*pan1-18TA*::*LEU2 ABP1-mCherry::URA3*

*HSE1-3GFP::HIS3 sac6* Δ*::KanMX* This study

JJTY4107 *Mat***a** *his3*-Δ*200* *leu2-3*, *112* *ura3-52* *pan1*Δ::*pan1-18TA*::*LEU2 ABP1-mCherry::URA3*

*HSE1-3GFP::HIS3 myo3*Δ::*KanMX myo5*Δ::*KanMX* This study

JJTY4133 *Mat***a** *his3*-Δ*200* *leu2-3*, *112* *ura3-52,* *lys2-801* *pan1*Δ::*pan1-18TA*::*LEU2*

*sla1*Δ:: *sla1-10TA*::LEU2 This study

JJTY4145 *Mat***a** *his3*-Δ*200* *leu2-3*, *112* *ura3-52,* *lys2-801* *pan1*Δ::*pan1-18TA*::*LEU2*

*sla1*Δ:: *sla1-10TA*::LEU2 *bar1*Δ:: *HIS3* This study

JJTY5485 *Mat***a** *his3-*Δ*200 lys2-801 pan*Δ*::pan1-18TA::LEU2 ABP140-3GFP::HIS PAN1-mCherry::URA* This study

JJTY5486 *Mat***α** *his3-*Δ* lys2-801 pan1-*Δ*pan1-18TA::LEU2 ABP1-mCherry::URA3 PAN1-GFP::HIS3* This study

JJTY5487 *Mat***a** *his3-*Δ*200 leu2-3,112 ura3-52 lys2-801 pan*Δ*::pan1-18TA::LEU2 HSE1-GFP::HIS* This study

*PAN1-mCherry::URA*

JJTY5644 *Mat***a** *his3*-Δ*200* *leu2-3*, *112* *ura3-52* *lys2-801* *pan1*Δ::*pan1-18TA*::*LEU2 PAN1-mCherry::URA3*

*HSE1-3GFP::HIS3 sac6* Δ*::KanMX* This study

JJTY5648 *Mat***a** *his3*-Δ*200* *leu2-3*, *112* *ura3-52 lys2-801 HSE1-GFP*::*HIS3 PAN1-mCherry::URA3*  This study

JJTY5919 *Mat***a** *his3*-Δ*200* *leu2-3*, *112* *ura3-52 ark1*Δ*::HIS3 prk1-as3::URA3 PAN1-mCherry::URA3* This study

*ABP140-3GFP::HIS3*

JJTY5920 *Mat***a** *his3*-Δ*200* *leu2-3*, *112* *ura3-52 lys2-801 pan1*Δ::*pan1-18TA* Δ855::*LEU2* This study

*ABP140-3GFP*::*HIS3 PAN1-mCherry::URA3*
