# Supplementary material for: Yeast Eps15-like endocytic protein Pan1p regulates the interaction between endocytic vesicles, endosomes and the actin cytoskeleton
Source: eLife. 2016 Feb 25;5:e10276. doi: 10.7554/eLife.10276 (PMC4775215; doi:10.7554/eLife.10276)
Supplement: Supplementary file 2. — DOI: http://dx.doi.org/10.7554/eLife.10276.024 [file elife-10276-supp2.docx]

**Supplementary file 2.** Primers used in this study

Primer Gene Sequence Use

JT373  *PAN1* CAACCCAACATTGCTGGCAATGCTT mutagenesis

JT372  *PAN1* AAGCATTGCCAGCAATGTTGGGTTG mutagenesis

JT374  *PAN1* GATGCCACAGACAGCTGGTATGATGCCACAGACAGCTGGTATGATGCC mutagenesis

JT375  *PAN1* GGCATCATACCAGCTGTCTGTGGCATCATACCAGCTGTCTGTGGCATC mutagenesis
